# Optimal Number of Lymph Nodes Retrieved to Lower the Risk of False N0 for Patients with Pancreatic Cancer Undergoing Curative Surgery

**DOI:** 10.1245/s10434-025-18029-7

**Published:** 2025-08-23

**Authors:** So Jeong Yoon, Seung Soo Hong, Boram Park, Sung Hyun Kim, Chang Moo Kang, Kyung Sik Kim, Sang Hyun Shin, In Woong Han, Jin Seok Heo, Ho Kyoung Hwang, Hongbeom Kim

**Affiliations:** 1 Division of Hepatobiliary-Pancreatic Surgery, Department of Surgery, Samsung Medical Center, Sungkyunkwan University School of Medicine, Seoul, Korea; 2 Division of Hepatobiliary and Pancreatic Surgery, Department of Surgery, Severance Hospital, Yonsei University College of Medicine, Seoul, Korea; 3 College of Medicine, Inha University, Incheon, Republic of Korea

## Abstract

**Background:**

Accurate LN examination is critical for staging and prognosis in pancreatic cancer. However, the ideal number of LNs required for precise staging and improved survival remains unclear. This study aimed to determine the optimal number of lymph nodes (LNs) to retrieve during pancreatectomy for pancreatic cancer to minimize false node-negative (false N0) rates and assess its impact on survival outcomes.

**Methods:**

This retrospective cohort study analyzed data from patients undergoing curative-intent upfront surgery for pancreatic cancer at two tertiary centers in South Korea (2010–2021). An exploration cohort of 808 patients was used to identify LN retrieval thresholds, and the results were validated in an independent cohort of 444 patients. The study excluded patients who received neoadjuvant therapy or had fewer than two retrieved LNs. False N0 rates and 5 year overall survival were analyzed.

**Results:**

In the exploration cohort, examining 16 LNs reduced the false N0 rate to 18.9%, whereas in the validation cohort, retrieving 12 LNs resulted in a false N0 rate of 19.5%. Among the node-negative (N0) patients, retrieving up to 21 LNs was associated with improved 5 year overall survival. Differences in cutoff values between cohorts were attributed to demographic variations and inclusion of fewer LNs retrieved but higher detection of metastatic nodes in the validation cohort.

**Conclusions:**

Retrieving a sufficient number of LNs during pancreatectomy is essential to reducing false N0 rates and improving survival outcomes for pancreatic cancer patients. These findings highlight the need for standardized LN evaluation protocols and support further prospective, multi-center studies to optimize staging accuracy.

Pancreatic ductal adenocarcinoma (PDAC) is one of the most aggressive malignancies, characterized by poor prognosis and limited long-term survival. Among several prognostic factors, lymph node (LN) status significantly influences disease progression and guides postoperative treatment strategies.^1,2^ Accurate staging of LN involvement is crucial because it informs prognosis and impacts adjuvant therapy decisions.

Current guidelines, such as those from the National Comprehensive Cancer Network (NCCN), recommend the retrieval and examination of at least 12 LNs during pancreatectomy for pancreatic cancer.^3^ However, evidence suggests that retrieving more LNs may further improve staging accuracy by reducing the risk of false node-negative (false N0) diagnoses.^4,5^ Although the NCCN guidelines provide a valuable baseline, the optimal number of LNs to be examined to minimize staging errors and maximize prognostic reliability remains a topic of debate, emphasizing the need for further research to refine these recommendations.

This study aimed to identify the optimal number of LNs to be retrieved and examined during pancreatectomy for pancreatic cancer to mitigate the risk of false N0 diagnoses. By analyzing retrospective clinical data, we aimed to deliver evidence-based recommendations to enhance the accuracy of nodal staging and the prognostic stratification of patients after curative resection.

## Methods

### Study Population

#### Exploration Cohort

This study included patients who underwent curative-intent surgery for PDAC at Samsung Medical Center between 2010 and 2021 and excluded patients who received neoadjuvant treatment or underwent open-and-closure due to unexpectedly advanced-stage or unresectable disease. The institutional review boards of Samsung Medical Center (SMC 2023-07-025) and Severance Hospital, Yonsei University College of Medicine (YUHS 4-2024-0900) approved this study and waived the requirement for written informed consent from participants because the research posed minimal risk to subjects and lacked a basis for anticipation of objection to the agreement.

### Study Population

#### Validation Cohort

An independent validation cohort included patients who underwent upfront surgery for pancreatic cancer without neoadjuvant treatment at Severance Hospital between 2010 and 2021. The data collected included clinicopathologic information and survival outcomes. The findings from the exploration cohort were externally validated using this validation cohort.

### Perioperative Data and Survival Outcomes

Demographic information, clinical characteristics, operative outcomes, and pathologic reports were retrospectively reviewed. Pathologic staging was based on the eighth edition of the American Joint Committee on Cancer (AJCC) staging system.^6^ The number of harvested and positive LNs was documented. Data concerning recurrence were obtained from medical records. Recurrence was suspected when tumor marker elevation coincided with radiologic findings indicative of recurrence. Confirmation of suspected recurrence was attempted via positron emission tomography (PET)-computed tomography (CT) or biopsy when possible. Recurrence-free survival (RFS) was defined as the time from surgery to the diagnosis of recurrence, and overall survival (OS) was defined as the interval from surgery to death from any cause.

### Definition of False N0 Rate and Statistical Modeling

False N0 was defined as the probability of a node-positive patient being misclassified as node-negative due to inadequate lymph node retrieval and examination. To estimate the false N0 rate relative to the number of pathologically examined lymph nodes, we applied the methodology described by Gönen et al.^7^ and Robinson et al.^8^ This method assumes that each retrieved lymph node independently has a certain probability of harboring metastasis, and models the likelihood of missing metastatic nodes using a binomial distribution framework. Specifically, the false N0 rate at a given number of examined LNs was estimated by modeling the probability that no metastatic node would be detected despite the presence of metastatic nodal disease. The prevalence of nodal metastasis observed in the cohort was used as the baseline probability in the calculation. All analyses were performed under the assumption of independent detection probability per node, consistent with previous applications of this model.

### Statistical Analysis

Comparisons between patient groups were performed using Student’s *t* test for continuous variables and the chi-square test for categorical variables. A *p* value lower than 0.05 was considered statistically significant.

To investigate the impact that the number of retrieved LNs had on OS, a restricted cubic spline function was used to depict the 5 year OS. All statistical analyses were conducted using SAS (version 9.3; SAS Institute Inc., Cary, NC, USA) and R software (The R Foundation for Statistical Computing, Vienna, Austria).

## Results

The demographic and clinicopathologic data for the exploration cohort (*n* = 808) and the validation cohort (*n* = 444) were compared, and the results are presented in Table 1. The mean preoperative carbohydrate antigen 19-9 (CA 19-9) level was significantly higher in the exploration cohort than in the validation cohort (649.7 vs. 276.6 U/mL; *P* = 0.021). The prevalence of the American Society of Anesthesiologists (ASA) score of 3 or 4 was higher in the validation cohort than in the exploration cohort (47.9% vs. 12.9 %; *P* < 0.001). The patients in the validation cohort experienced a longer hospital stay (17.1 vs. 11.5 days; *P* < 0.001).

**Table 1:** Comparative analysis of demographic and clinicopathologic data between the exploration (*n* = 808) and validation (*n* = 444) cohorts

Variables	Exploration cohort (*n* = 808) *n* (%)	Validation cohort (*n* = 444) *n* (%)	*P* Value
Mean age (years)	65.3 ± 9.7	65.2 ± 9.3	0.883
Male sex	465 (57.5	249 (56.1)	0.592
Underlying DM	294 (36.4)	180 (40.5)	0.162
Mean preoperative CEA (ng/mL)	3.2 ± 4.7	3.6 ± 3.5	0.180
Mean preoperative CA 19-9 (U/mL)	649.7 ± 4441.6	276.6 ± 686.3	0.021
ASA score of 3 or 4	104 (12.9)	213 (47.9)	<0.001
Type of operation			0.720
Pancreaticoduodenectomy	465 (57.5)	261 (58.8)	
Left-sided pancreatectomy	343 (42.5)	183 (41.2)	
Mean estimated blood loss (mL)	370.8 ± 334.2	405.5 ± 628.7	0.283
Mean hospital stay (days)	11.5 ± 7.9	17.1 ± 20.9	<0.001
Pathologic stage (AJCC 8th)			<0.001
I	338 (41.8)	205 (46.2)	
II	415 (51.4)	173 (38.9)	
III	55 (6.8)	66 (14.9)	
T stage			0.046
T1	193 (23.9)	112 (25.2)	
T2	506 (62.6)	293 (66.0)	
T3	109 (13.5)	39 (8.8)	
N stage			<0.001
N0	370 (45.8)	220 (49.5)	
N1	383 (47.4)	158 (35.6)	
N2	55 (6.8)	66 (14.9)	
Mean no. of examined LNs	18.2 ± 10.1	16.6 ± 10.8	0.011
Mean no. of metastatic LNs	1.3 ± 2.1	1.5 ± 2.4	0.131
Adjuvant treatment	545 (69.2)	363 (81.8)	<0.001
Median recurrence-free survival (months)	14.0	16.6	0.432
Median overall survival (months)	29.0	35.8	0.022

DM, diabetes mellitus; CEA, carcinoembryonic antigen; CA19-9, carbohydrate antigen 19-9; ASA, American Society of Anesthesiologists; AJCC, American Joint Committee on Cancer; LN, lymph node

In the pathology report, the number of examined LNs was greater in the exploration cohort than in the validation cohort (18.2 vs. 16.6; *P* = 0.011). Statistically significant differences were observed in the proportion of patients regarding pathologic stage, T stage, and N stage. A higher percentage of patients in the validation cohort received adjuvant treatment than in the exploration cohort (82.3% vs. 69.2%; *P* < 0.001). The median OS was significantly longer in the validation cohort than in the development cohort (35.8 vs. 29.0 months; *P* = 0.022).

**Fig. 1:**
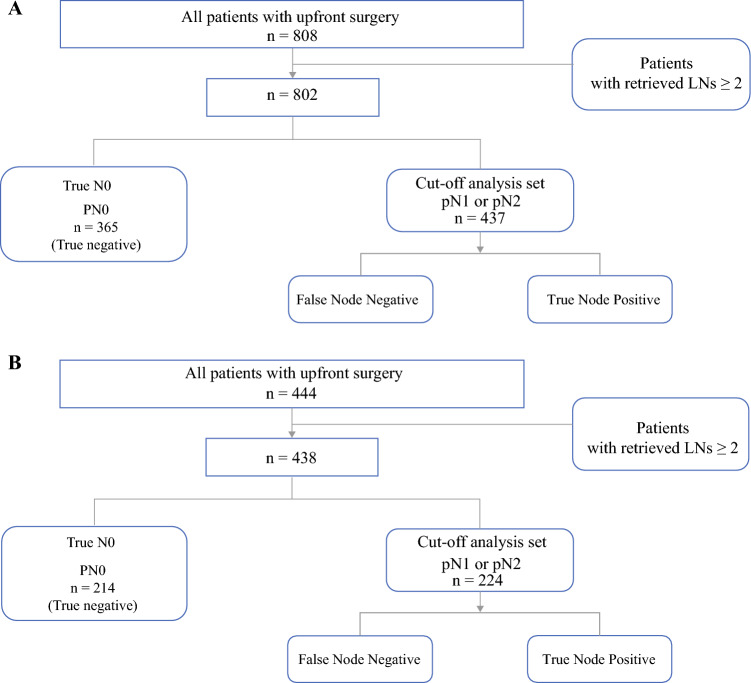
Patient selection in the **A** exploration and **B** validation cohorts

Figure 1 illustrates the patient selection process for cutoff-point analysis performed for both the exploration (Fig. 1A) and validation (Fig. 1B) cohorts. The inclusion criteria for the analysis specified patients with at least two retrieved and pathologically examined LNs. The patients with at least one metastatic LN (pN1 or pN2) were included in the cutoff analysis sets, assuming the possibility of false N0 among these patients. Finally, the cutoff analysis included 437 patients in the exploration cohort and 224 patients in the validation cohort.

The comparisons between the cutoff analysis sets from exploration (*n* = 437) and validation (*n* = 224) cohorts are shown in Table 2. Diabetes mellitus (DM) was more prevalent in the validation cohort than in the exploration cohort (41.1% vs. 33.2%; *P* = 0.049). The validation cohort also had a significantly higher proportion of patients with an ASA score of 3 or 4 (47.8% vs. 11.4%; *P* < 0.001) and longer hospital stays (17.3 vs. 12.3 days; *P* < 0.001). Differences in pathologic stage were notable, with more patients having stage III and N2 disease in the validation cohort than in the exploration cohort (29.5% vs. 12.6%; *P* < 0.001 for both). The number of retrieved and pathologically examined LNs did not differ significantly between the two sets (20.7 vs. 19.4; *P* = 0.138). However, the number of metastatic LNs was higher in the validation cohort (3.0 ± 2.7 vs. 2.4 ± 2.4; *P* = 0.006). More patients in the validation cohort received adjuvant treatment than in the exploration cohort (82.4% vs. 72.4%; *P* = 0.005). No significant differences in survival were noted between the two groups.

**Table 2:** Comparative analysis of demographic and clinicopathologic data for the cutoff point analysis sets

Variables	Exploration set (*n* = 437) *n* (%)	Validation set (*n* = 224) *n* (%)	*P* Value
Mean age (years)	64.4 ± 9.5	64.4 ± 9.7	0.976
Sex, male	261 (59.7)	129 (57.6)	0.617
Underlying DM	145 (33.2)	92 (41.1)	0.049
Mean preoperative CEA (ng/mL)	3.4 ± 4.9	3.9 ± 4.0	0.165
Mean preoperative CA 19-9 (U/mL)	923.7 ± 5981.2	396.2 ± 915.8	0.076
ASA score of 3 or 4	50 (11.4)	107 (47.8)	<0.001
Type of operation			0.233
Pancreaticoduodenectomy	269 (61.6)	149 (66.5)	
Left-sided pancreatectomy	168 (38.4)	75 (33.5)	
Mean estimated blood loss (mL)	395.6 ± 915.8	460.7 ± 816.9	0.262
Mean hospital stay (days)	12.3 ± 99	17.3 ± 15.0	<0.001
Pathologic stage (AJCC 8th)			<0.001
I	0	0	
II	382 (87.4)	158 (70.5)	
III	55 (12.6)	66 (29.5)	
T stage			0.076
T1	64 (14.6)	37 (16.5)	
T2	297 (68.0)	163 (72.8)	
T3	76 (17.4)	24 (10.7)	
N stage			<0.001
N0	0	0	
N1	382 (87.4)	158 (70.5)	
N2	55 (12.6)	66 (29.5)	
Mean no. of examined LNs	20.7 ± 10.0	19.4 ± 10.5	0.138
Mean no. of metastatic LNs	2.4 ± 2.4	3.0 ± 2.7	0.006
Adjuvant treatment	310 (72.4)	183 (81.7)	0.005
Median recurrence-free survival (months)	11.0	11.4	0.566
Median overall survival (months)	22.0	25.0	0.225

DM, diabetes mellitus; CEA, carcinoembryonic antigen; CA19-9, carbohydrate antigen 19-9; ASA, American Society of Anesthesiologists; AJCC, American Joint Committee on Cancer; LN, lymph node

**Fig. 2:**
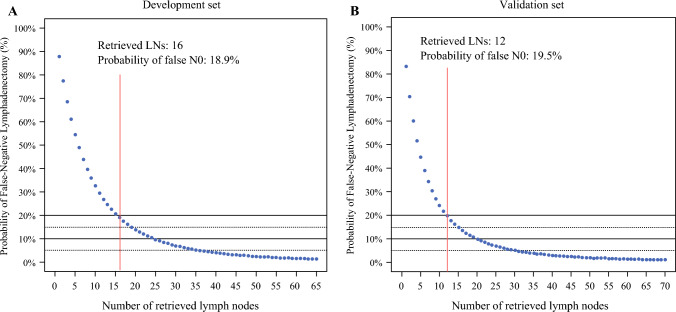
Assessment of false N0 likelihood based on the number of retrieved lymph nodes in the **A** exploration and **B** validation sets

Figure 2 illustrates the false N0 rate relative to the number of pathologically examined LNs in both the exploration and validation sets. Detailed false N0 rates for each LN count are available in Tables 3 and 4, respectively. In the exploration set, retrieving 15 LNs resulted in a false N0 rate of 20.5%, whereas dissecting 16 LNs reduced the false N0 rate further to 18.9%. In the validation set, retrieving 12 LNs reduced the false N0 rate to 19.5%, and retrieving 16 LNs further decreased it significantly to 13.4%.

**Table 3:** False N0 rates according to the number of examined lymph nodes in the exploration set (*n* = 437)

Total no. of examined LNs	False N0 rate (%)	Total no. of examined LNs	False N0 rate (%)	Total no. of examined LNs	False N0 rate (%)
1	87.8	16	18.9	31	6.4
2	77.5	17	17.4	32	6.0
3	68.6	18	16.0	33	5.7
4	61.0	19	14.8	34	5.4
5	54.5	20	13.7	35	5.1
6	48.8	21	13.7	36	4.8
7	43.8	22	12.7	37	4.5
8	39.5	23	11.8	38	4.3
9	35.7	24	11.0	39	4.0
10	32.4	25	10.2	40	3.8
11	29.4	26	9.5	41	3.6
12	26.8	27	8.9	42	3.4
13	24.5	28	8.3	43	3.3
14	22.4	29	7.8	44	3.1
15	20.5	30	7.3	45	3.0

LN, lymph node

**Table 4:** False N0 rates according to the number of examined lymph nodes in the validation set (*n* = 224)

Total no. of examined LNs	False N0 rate (%)	Total no. of examined LNs	False N0 rate (%)	Total no. of examined LNs	False N0 rate (%)
1	83.3	16	13.4	31	4.6
2	70.3	17	12.3	32	4.4
3	59.9	18	11.3	33	4.1
4	51.5	19	10.4	34	3.9
5	44.6	20	9.7	35	3.7
6	39.0	21	8.9	36	3.5
7	34.3	22	8.3	37	3.3
8	30.3	23	7.7	38	3.2
9	27.0	24	7.2	39	3.0
10	24.1	25	6.7	40	2.9
11	21.7	26	6.3	41	2.7
12	19.5	27	5.9	42	2.6
13	17.7	28	5.5	43	2.5
14	16.1	29	5.2	44	2.4
15	14.7	30	4.9	45	2.3

LN, lymph node

**Fig. 3:**
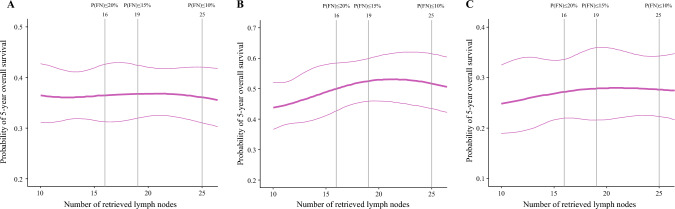
Linear relationship between the number of examined lymph nodes and the 5 year survival probability across both the exploration and validation cohorts: **A** in the patients overall, **B** in the N0 patients, and **C** in the N1/N2 patients

The linear relationship between the total number of examined LNs and the 5 year OS probability is demonstrated in Fig. 3, based on an integrated analysis of the exploration and validation cohorts (*n* = 1252). No significant survival trends were observed in the overall patient population (Fig. 3A). However, among the N0 patients (Fig. 3B), survival probability consistently improved with an increase in the number of examined LNs up to 21, with 16 LNs serving as an intermediate point. Among the N1 and N2 patients (Fig. 3C), a similar trend of improved OS with an increasing number of examined LNs was noted, although it was less pronounced than among the N0 patients.

## Discussion

Nodal status is a crucial prognostic factor in PDAC, affecting both OS and disease progression.^1,2^ This study sought to determine the probability of false N0 status in PDAC based on the number of retrieved and pathologically examined LNs. A comprehensive review of more than 800 consecutive cases in the exploration cohort was conducted to explore the relationship between the specific number of examined LNs and the false N0 rate. These findings were subsequently validated using an independent cohort. Additionally, the survival trend based on the total number of pathologically examined nodes also was investigated.

Accurate nodal staging in PDAC has significant clinical implications. As a key component of the AJCC cancer staging system,^6^ nodal status plays a pivotal role in determining prognosis. A recent meta-analysis identified pathologic nodal status as a key prognostic factor for early recurrence (within 12 months after surgery), together with factors such as the absence of adjuvant chemotherapy and elevated preoperative CA 19-9 levels.^9^ Moreover, a previous study from our institution demonstrated that LN metastasis had a higher odds ratio for predicting 5 year recurrence-free survival than tumor size, differentiation, or resection margin status.^10^ In addition to its prognostic value, LN status is essential for guiding adjuvant treatment strategies because it may influence the efficacy of adjuvant treatment.^11^

Another study reported that patients with metastatic LNs responded better to oxaliplatin, irinotecan, leucovorin, and fluorouracil (FOLFIRINOX)-based adjuvant therapy than those with node-negative disease.^12^ Concerning additional radiotherapy, Liu et al.^13^ demonstrated that adjuvant chemoradiotherapy after resection of pancreatic cancer leads to survival improvement only for patients with node-positive disease. Taken together, precise nodal evaluation, which decreases the false N0 rate, is of critical importance in optimizing therapeutic outcomes for patients with resected PDAC.

Regarding the adequate number of LNs retrieved during pancreatectomy for PDAC, previous studies have proposed various thresholds for accurate staging. Notably, the NCCN guidelines recommend retrieving at least 12 LNs,^3^ whereas the International Study Group on Pancreatic Surgery (ISGPS) suggests a minimum of 15 LNs for patients without neoadjuvant treatment.^14^ In 2019, Arrington et al.^15^ recommended sampling 11 to 17 LNs and suggested that 18 LNs are required to capture 90% of node-positive disease.^15^ Hua et al.^16^ set LN thresholds based on T stage, recommending examination of at least 16, 21, and 23 LNs respectively for T1, T2, and T2 tumors.

Our study aimed to reduce the false N0 rate to 20% identifying thresholds of 16 LNs in the exploration cohort. Importantly, this finding was validated in an independent validation cohort, in which examination of 16 LNs was associated with a false N0 rate of 13.4%, confirming the robustness of our threshold across different patient populations. Although these thresholds are somewhat consistent with previous recommendations, our study uniquely correlated the number of retrieved LNs with quantitatively derived false N0 rates across two independent cohorts. Furthermore, by linking LN retrieval numbers to 5 year OS outcomes, we extended the clinical significance beyond staging accuracy alone. To facilitate further multicenter studies and to improve staging accuracy, development of a standardized protocol for nodal status evaluation is needed.

The difference in cutoff values between the exploration and validation sets could be attributed to subtle differences in patient and procedural characteristics. Notably, although baseline tumor markers such as CA 19-9 and carcinoembryonic antigen (CEA) did not differ significantly between the two sets, a higher proportion of patients in the exploration set had more advanced pathologic stages of disease. More complex cases may have required more extensive resections, potentially leading to a higher baseline expectation for LN retrieval. Despite this, the number of pathologically examined lymph nodes in the exploration set was not markedly lower than in the validation set.

All surgeries in both cohorts were performed under the assumption of standardized lymphadenectomy by experienced surgeons. Therefore, the observed differences in LN yield and cutoff values may reflect institutional variability in the gross handling and pathologic processing of surgical specimens rather than true differences in surgical technique. These findings underscore the need to standardize not only surgical approaches but also pathologic evaluation protocols across institutions to ensure reproducible LN staging.

Numerous studies have demonstrated a potential correlation between the number of retrieved LNs and survival outcomes in PDAC. Foundational studies by Slidell et al.^17^ and Schwarz and Smith,^18^ based on large national databases, have established that both the total number of retrieved LNs and the extent of lymphadenectomy are independently associated with improved survival and staging accuracy in pancreatic cancer.

Some studies have further reported a direct relationship between the number of retrieved LNs and survival. For example, Contreras et al.^19^ analyzed the National Cancer Data Base (NCDB) in the United States and confirmed that LN retrieval is positively correlated with survival. Another study^20^ using the Surveillance, Epidemiology, and End Results (SEER) database reported that the total number of harvested LNs is a significant protective factor for survival, even for patients with N0 disease. Other studies emphasized the potential prognostic importance of retrieving and examining an adequate number of LNs because it enables more accurate staging and reduces the risk of undertreating node-positive patients. Prassas et al.^21^ underscored the need for sufficient LN harvest for applying nodal classification systems effectively. Additionally, Tarantino et al.^22^ demonstrated that the number of positive lymph nodes itself provides strong prognostic stratification in PDAC, reinforcing the role of detailed nodal assessment.

In the context of these studies, our findings further substantiate the importance of LN retrieval and examination in determining survival outcomes. We observed that retrieving up to 21 LNs was associated with improved 5 year survival, particularly for node-negative (N0) patients. This trend was less pronounced but still evident for node-positive (N1 and N2) patients, and the cutoff values of 16 and 12 LNs identified in our exploration and validation sets, respectively, fall within this range. However, the underlying mechanism remains uncertain. It is unclear whether this association reflects improved staging accuracy, by minimizing false N0 cases, or a potential therapeutic benefit from extended lymphadenectomy itself. Given the retrospective nature of our study and variability in pathologic practices, causal inference is limited. We believe that efforts to standardize both surgical lymphadenectomy and pathologic examination are necessary preconditions to enable in-depth analysis of the relationship between LN retrieval and survival. Future prospective studies should be designed for further investigation of this distinction.

Several limitations of our study must be acknowledged. First, the retrospective nature of the study introduced inherent biases, including potential variability in data collection and documentation. This may impact the generalizability of our findings despite efforts to ensure robust statistical analysis.

Second, the study involved multiple surgeons performing pancreatectomies at the same institution during a decade. Variations in surgical technique and LN dissection practices among surgeons could have influenced the number of retrieved LNs and the subsequent pathologic evaluation.

Third, differences in gross specimen-handling by pathologists could have contributed further to variability in LN counts. Additionally, institutional protocols for pre- and postoperative management, including adjuvant treatment and surveillance, may vary based on individual expertise and practices, potentially impacting the consistency of our data.

These limitations underscore the need for standardized surgical and pathologic protocols in future prospective studies to validate our findings and improve generalizability. Despite these challenges, our study provides practical guidance for optimizing LN examination and staging in PDAC, paving the way for more accurate prognostic assessments and improved patient outcomes. Moreover, this dual-cohort approach strengthens the reliability of our findings.

In conclusion, we proposed and validated cutoff numbers of LNs for examination to reduce the false N0 rate in PDAC. By referencing these specific thresholds, clinicians can minimize the underestimation of LN metastasis, providing a potential foundation for improving oncologic outcomes for patients with resected PDAC.

## References

[1] House MG, Gonen M, Jarnagin WR, et al. Prognostic significance of pathologic nodal status in patients with resected pancreatic cancer. *J Gastrointest Surg*. 2007;11:1549–55. 10.1007/s11605-007-0243-7. 17786531 10.1007/s11605-007-0243-7

[2] You MS, Lee SH, Choi YH, et al. Lymph node ratio as valuable predictor in pancreatic cancer treated with R0 resection and adjuvant treatment. *BMC Cancer*. 2019;19:952. 10.1186/s12885-019-6193-0. 31615457 10.1186/s12885-019-6193-0 PMC6794802

[3] National Comprehensive Cancer Network. NCCN Clinical Practice Guidelines in Oncology (NCCN Guidelines^®^) for Pancreatic Adenocarcinoma V.3.2024. Retrieved 1 December 1 2024 at https://www.nccn.org/Guidelines/Guidelines-Detail?Category=1&Id=1455.

[4] Huang L, Jansen L, Balavarca Y, et al. Significance of examined lymph node number in accurate staging and long-term survival in resected stage I-II pancreatic cancer–more is better? A large international population-based cohort study. *Ann Surg*. 2021;274:e554–63. 10.1097/sla.0000000000003558. 31425290 10.1097/SLA.0000000000003558

[5] Huebner M, Kendrick M, Reid-Lombardo KM, et al. Number of lymph nodes evaluated: prognostic value in pancreatic adenocarcinoma. *J Gastrointest Surg*. 2012;16:920–6. 10.1007/s11605-012-1853-2. 22421988 10.1007/s11605-012-1853-2

[6] Amin MB, Edge S, Greene F, et al. AJCC cancer staging manual. 8th edn. New York: Springer International Publishing; 2017.

[7] Gönen M, Schrag D, Weiser MR. Nodal staging score: a tool to assess adequate staging of node-negative colon cancer. *J Clin Oncol*. 2009;27:6166–71. 10.1200/jco.2009.23.7958. 19901106 10.1200/JCO.2009.23.7958 PMC3651597

[8] Robinson TJ, Thomas S, Dinan MA, Roman S, Sosa JA, Hyslop T. How many lymph nodes are enough? Assessing the adequacy of lymph node yield for papillary thyroid cancer. *J Clin Oncol*. 2016;34:3434–9. 10.1200/jco.2016.67.6437. 27528716 10.1200/JCO.2016.67.6437 PMC6366339

[9] Leonhardt CS, Gustorff C, Klaiber U, et al. Prognostic factors for early recurrence after resection of pancreatic cancer: a systematic review and meta-analysis. *Gastroenterology*. 2024;167:977–92. 10.1053/j.gastro.2024.05.028. 38825047 10.1053/j.gastro.2024.05.028

[10] Yoon SJ, Shin SH, Yoon SK, et al. Appraisal of 5-year recurrence-free survival after surgery in pancreatic ductal adenocarcinoma. *J Hepatobiliary Pancreat Sci*. 2021;28:287–96. 10.1002/jhbp.815. 32790012 10.1002/jhbp.815

[11] Liu C, Cheng H, Jin K, et al. Resected pancreatic cancer with N2 node involvement is refractory to gemcitabine-based adjuvant chemotherapy. *Cancer Control*. 2020;27:1073274820915947. 10.1177/1073274820915947. 32268796 10.1177/1073274820915947 PMC7153189

[12] Conroy T, Hammel P, Hebbar M, et al. FOLFIRINOX or gemcitabine as adjuvant therapy for pancreatic cancer. *N Engl J Med*. 2018;379:2395–406. 10.1056/NEJMoa1809775. 30575490 10.1056/NEJMoa1809775

[13] Liu Z, Luo G, Guo M, et al. Lymph node status predicts the benefit of adjuvant chemoradiotherapy for patients with resected pancreatic cancer. *Pancreatology*. 2015;15:253–8. 10.1016/j.pan.2015.03.012. 25921232 10.1016/j.pan.2015.03.012

[14] Tol JA, Gouma DJ, Bassi C, et al. Definition of a standard lymphadenectomy in surgery for pancreatic ductal adenocarcinoma: a consensus statement by the International Study Group on Pancreatic Surgery (ISGPS). *Surgery*. 2014;156:591–600. 10.1016/j.surg.2014.06.016. 25061003 10.1007/978-1-4939-1726-6_59 PMC7120678

[15] Arrington AK, Price ET, Golisch K, Riall TS. Pancreatic cancer lymph node resection revisited: a novel calculation of number of lymph nodes required. *J Am Coll Surg*. 2019;228:662–9. 10.1016/j.jamcollsurg.2018.12.031. 30677528 10.1016/j.jamcollsurg.2018.12.031

[16] Hua J, Zhang B, Xu J, et al. Determining the optimal number of examined lymph nodes for accurate staging of pancreatic cancer: an analysis using the nodal staging score model. *Eur J Surg Oncol*. 2019;45:1069–76. 10.1016/j.ejso.2019.01.018. 30685327 10.1016/j.ejso.2019.01.018

[17] Slidell MB, Chang DC, Cameron JL, et al. Impact of total lymph node count and lymph node ratio on staging and survival after pancreatectomy for pancreatic adenocarcinoma: a large, population-based analysis. *Ann Surg Oncol*. 2008;15:165–74. 10.1245/s10434-007-9587-1. 17896141 10.1245/s10434-007-9587-1

[18] Schwarz RE, Smith DD. Extent of lymph node retrieval and pancreatic cancer survival: information from a large US population database. *Ann Surg Oncol*. 2006;13:1189–200. 10.1245/s10434-006-9016-x. 16955385 10.1245/s10434-006-9016-x

[19] Contreras CM, Lin CP, Oster RA, et al. Increased pancreatic cancer survival with greater lymph node retrieval in the National Cancer Data Base. *Am J Surg*. 2017;214:442–9. 10.1016/j.amjsurg.2017.06.036. 28687101 10.1016/j.amjsurg.2017.06.036 PMC5576522

[20] Hellan M, Sun CL, Artinyan A, et al. The impact of lymph node number on survival in patients with lymph node-negative pancreatic cancer. *Pancreas*. 2008;37:19–24. 10.1097/MPA.0b013e31816074c9. 18580439 10.1097/MPA.0b013e31816074c9

[21] Prassas D, Safi SA, Stylianidi MC, et al. N, LNR or LODDS: Which is the most appropriate lymph node classification scheme for patients with radically resected pancreatic cancer? *Cancers Basel*. 2022;14:1834. 10.3390/cancers14071834. 35406606 10.3390/cancers14071834 PMC8997819

[22] Tarantino I, Warschkow R, Hackert T, et al. Staging of pancreatic cancer based on the number of positive lymph nodes. *Br J Surg*. 2017;104:608–18. 10.1002/bjs.10472. 28195303 10.1002/bjs.10472

